# Multifunctional Albumin-Stabilized Gold Nanoclusters for the Reduction of Cancer Stem Cells

**DOI:** 10.3390/cancers11070969

**Published:** 2019-07-10

**Authors:** Ana Latorre, Alfonso Latorre, Milagros Castellanos, Ciro Rodriguez Diaz, Ana Lazaro-Carrillo, Tania Aguado, Mercedes Lecea, Sonia Romero-Pérez, Macarena Calero, José María Sanchez-Puelles, Ángeles Villanueva, Álvaro Somoza

**Affiliations:** 1 Instituto Madrileño de Estudios Avanzados en Nanociencia (IMDEA Nanociencia) & Nanobiotecnología (IMDEA-Nanociencia), Unidad Asociada al Centro Nacional de Biotecnología (CSIC), 28049 Madrid, Spain; 2 Departamento de Biología, Facultad de Ciencias, Universidad Autónoma de Madrid, 28049 Madrid, Spain; 3 Centro de Investigaciones Biológicas, Consejo Superior de Investigaciones Científicas, 28040 Madrid, Spain

**Keywords:** nanomedicine, drug delivery, stimuli-responsive, DOX, SN38, CSCs

## Abstract

Controlled delivery of multiple chemotherapeutics can improve the effectiveness of treatments and reduce side effects and relapses. Here in, we used albumin-stabilized gold nanoclusters modified with doxorubicin and SN38 (AuNCs-DS) as combined therapy for cancer. The chemotherapeutics are conjugated to the nanostructures using linkers that release them when exposed to different internal stimuli (Glutathione and pH). This system has shown potent antitumor activity against breast and pancreatic cancer cells. Our studies indicate that the antineoplastic activity observed may be related to the reinforced DNA damage generated by the combination of the drugs. Moreover, this system presented antineoplastic activity against mammospheres, a culturing model for cancer stem cells, leading to an efficient reduction of the number of oncospheres and their size. In summary, the nanostructures reported here are promising carriers for combination therapy against cancer and particularly to cancer stem cells.

## 1. Introduction

Besides surgery, chemotherapy is the most common approach to treat cancer. However, this strategy is far from ideal due to the side effects caused by the toxicity of the chemotherapeutics employed. Moreover, multidrug resistance (MDR) [1] is developed when a single drug is administered multiple times, reducing the efficiency of the treatments. To overcome these limitations, chemotherapy is administered as a combination of two or more drugs, which synergistic effect [2] reduces the development of MDR. Furthermore, drug-related toxicity can be reduced due to the lower doses of the individual chemotherapeutic drugs employed in this approach [3]. However, the different solubility, pharmacokinetics, and biodistribution of the therapeutic agents may prevent their accumulation in the required concentrations at the tumor site, reducing their efficacy against the disease [4].

On the other hand, using carriers to deliver drugs has been shown to improve their efficiency and reduce their side effects [5]. This is mainly achieved by evading the reticuloendothelial system, which improves the pharmacokinetic properties of the drugs. In addition, those carriers with a nanometer size larger than 10 nm present an additional advantage due to the enhanced permeability and retention effect (EPR) [6], which leads to preferential accumulation of the delivered drug at the tumor area. Below that size, nanoparticles are quickly eliminated by the kidneys, reducing their interaction with the tumoral area [7]. As a consequence, the efficiency of the treatment may be increased, and the side effects reduced. In this regard, gold nanoclusters stabilized with bovine serum albumin have shown remarkable results in cell culture [8] and animal models [9,10]. This material accumulates efficiently in the tumor area, and therefore it has been used successfully in drug delivery [11,12]. In addition, gold nanoclusters present excellent fluorescent properties, and many reports are focused on exploiting that for imaging purposes [13,14]. The use of serum albumin as a coating for nanostructures provides excellent biochemical and biophysical properties in the in vivo experiments [15]. Furthermore, this coating contains amino and carboxy groups that facilitate the conjugation of different active molecules, such as, small molecule ligands [16], antibodies [17], or drugs [11,12], providing a convenient platform for preparing tailored nanostructures for efficient therapy.

Despite the excellent properties of albumin-stabilized gold nanoclusters, the use of this nanomaterial to deliver more than one drug has not been explored. Even reports on albumin conjugates containing more than one drug are scarce [18]. For these reasons, we decided to prepare a conjugate of albumin-stabilized gold nanoclusters containing two chemotherapeutic drugs for combined therapy.

Particularly, for the functionalization of the albumin-stabilized gold nanoclusters, we selected Doxorubicin (DOX) and SN38. DOX is a first line chemotherapeutic agent widely used in different kind of cancers. It is a hydrophilic molecule that binds topoisomerase II or intercalates in the DNA causing apoptosis [19] or other cell death mechanism depending on DOX concentration [20]. On the other hand, the camptothecin (CPT) analog SN38, is a potent topoisomerase I inhibitor [21], whose use in chemotherapy is limited because of its poor pharmacokinetic and high hydrophobicity [22]. Although the combination of DOX and camptothecins (CPTs) analogs have shown poor results in clinical trials, the pair DOX-CPT has been recently reported as one of the most synergistic combinations when co-delivered as a polymer-drug conjugate [23]. In addition, we envisioned that this combination might also be effective against the tumor-initiating cells, also known as Cancer Stem Cells (CSCs). These cells are a promising target in modern drug discovery programmes [24], since this subpopulation of malignant cells has an optimized DNA repair system, which is responsible for its resistance to therapy. Indeed, the combination of chemotherapeutics has been shown to reduce the population of CSCs [25,26], but this combination of drugs or this nanomaterial [27] has not been reported.

Our results show that the system combining both drugs presents excellent antitumor activity in different cancer cell models, including mammospheres, confirming the promising potential of this nanotherapeutic.

## 2. Results

### 2.1. Characterization of Functionalized AuNCs

**Figure 1:**
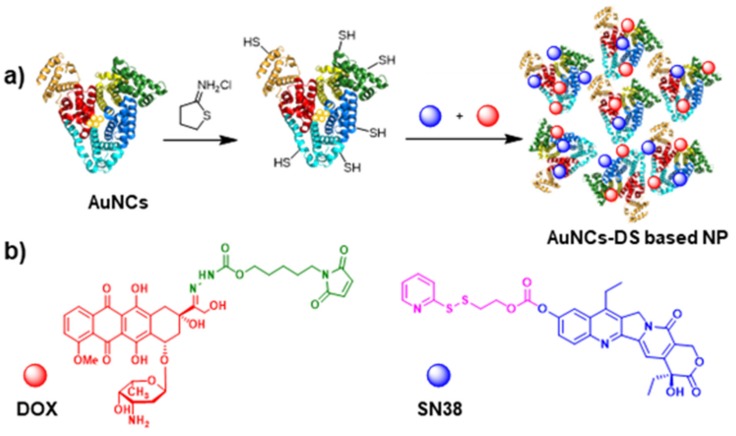
Synthesis of nanoparticles based on AuNCs modified with Doxorubicin (DOX) and SN38. (**a**) Schematic representation of the synthesis of DOX/SN38-AuNCs-based nanoparticles for combined chemotherapy; (**b**) Representation of DOX (red) modified with a pH-sensitive linker (green) and SN38 (blue) modified with a redox-sensitive linker (pink).

The albumin-based nanoparticles were prepared by the incubation of BSA with a gold salt and NaOH. This process leads to the formation of the corresponding AuNCs stabilized by BSA. The functionalization of the structure with the drugs requires the introduction of thiols in the structure using iminothiolane. Then, the drugs modified with linkers were added to the nanostructures (Figure 1). In the case of SN38, a linker sensitive to the reductive environment (e.g., GSH) was used. On the other hand, DOX was modified with a different linker sensitive to acidic pHs. Both systems contain a moiety that eases the conjugation with the thiol groups, a disulfide and a maleimide, respectively. Using these derivatives, the AuNCs stabilized by BSA were modified with DOX (AuNCs-D), SN38 (AuNCs-S), or both (AuNCs-DS).

The incorporation of DOX and SN38 on the AuNCs was studied by UV-VIS (Figure S1) after the removal of unbound material. The UV-Vis spectra of AuNCs-D revealed the standard absorption profile of AuNCs and the characteristic band of DOX centered at 495 nm. With AuNCs-S, a band centered at 380 nm corresponding to the absorption of SN38 could be identified. Finally, UV-VIS spectra of AuNCs-DS evidenced the bands corresponding to the three components, indicating that both drugs, DOX and SN38, were efficiently attached to AuNCs. The concentration of the drugs was obtained by interpolating the absorbance measured at 495 nm for DOX and 380 nm for SN38 in the corresponding calibration curve, obtaining 37 µM and 80 µM, respectively. In the case of bi-functionalized nanoclusters AuNCs-DS, the concentrations of DOX and SN38 were 37 µM and 70 µM, respectively.

**Figure 2:**
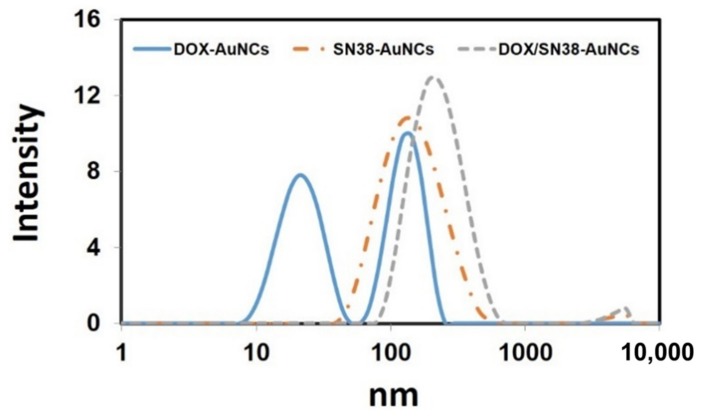
Size of Doxorubicin (DOX)-AuNCs, SN38-AuNCs and DOX/SN38-AuNCs measured by dynamic light scattering (DLS).

The sizes of the structures obtained were studied by dynamic light scattering (DLS) and SEM. DLS measurements of the AuNCs-D revealed the formation of a non-homogeneous material with two nanoparticle size distributions (Figure 2). However, when the hydrophobic SN38 was employed, monodispersed nanoparticles were obtained with an average size of 117.5 nm and a polydispersity index of 0.277. The combined use of DOX and SN38 does not disrupt the formation of monodispersed nanoparticles with an average size of 190.8 nm and a PDI of 0.263. In all cases, the structures obtained presented a globular shape when analyzed by SEM (Figure S2).

Interestingly, the nanoparticles bearing both drugs retained their colloidal stability and size in PBS at least over 15 days (Figure S3). Through this period, DOX remains essentially fully linked to the system, while SN38 is less stable and 62% of the drug is released.

**Figure 3:**
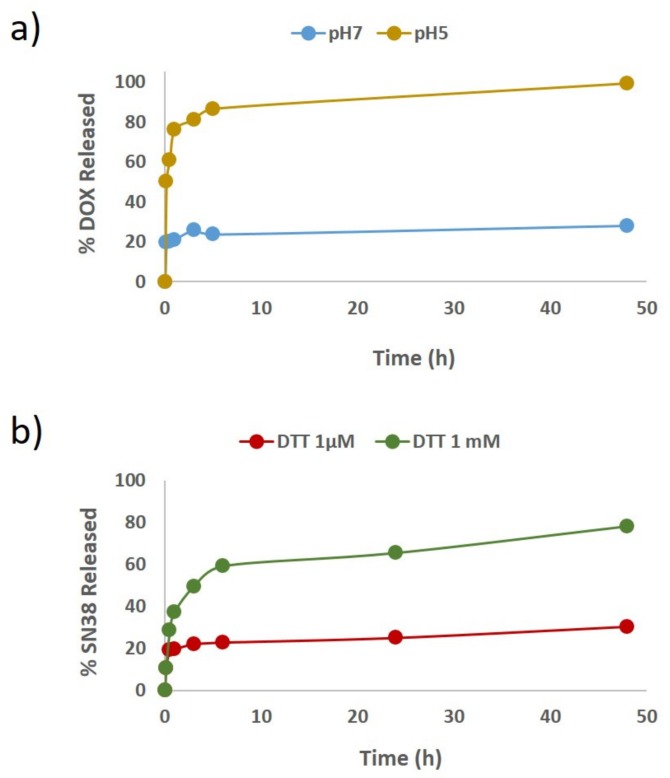
Release profile of Doxorubicin (DOX) and SN38 from AuNCs. (**a**) DOX release profile from AuNCs based nanoparticles in phosphate-citrate buffer (pH = 5 or 7), (**b**) SN38 release profile from AuNCs based nanoparticles in PBS containing 1 mM or 1 µM of DTT.

Then, the release of the chemotherapeutics in AuNCs-DS was studied in vitro under the selected triggering conditions. First, DOX release was studied by re-dissolving the nanoparticles in phosphate–citrate buffer at pH = 5. Under these acidic conditions, we observed that the ca. 85% of the total DOX conjugated was released within the first 5 h. Conversely, when the nanoparticles are maintained at pH = 7 the DOX release was not superior to 20%. SN38 release was studied in PBS containing DTT at a concentration of 1 mM, where 80% was released after 48 h. However, when micromolar concentration of DTT was employed, less than 30% was released at the same time (Figure 3a,b, respectively).

### 2.2. Chemotherapeutic Activity of Functionalized AuNCs in MCF7 Cells

**Figure 4:**
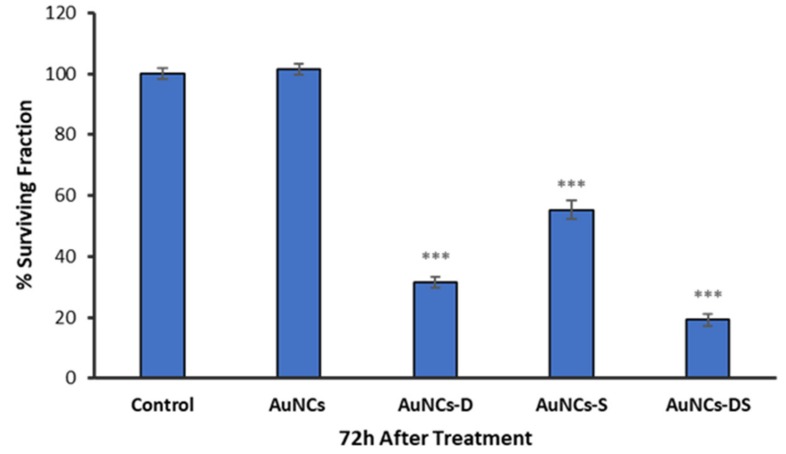
Surviving fraction of MCF-7 cells incubated 24 h with the different AuNCs formulations and evaluated 48 h after by MTT viability assay. Data correspond to mean ± S.D. values from at least six experiments. Statistical analysis was performed using one-way ANOVA Tukey’s test (each group vs. Control). *** *p* < 0.001.

In vitro toxicity studies of mono- and bi-functionalized AuNCs were performed in MCF-7 using a constant concentration of AuNCs of 2.6 µM. Cells were exposed to the nanostructures for 24 h, and the cell viability was determined after 48 h. As shown in Figure 4, no significant differences in cell viability were detected in samples exposed to non-functionalized AuNCs compared to untreated samples. This result highlights the excellent biocompatibility of this material. In contrast, mono-functionalized nanoparticles induced a significant reduction in cell survival, being AuNCs-D (70% cell death) more cytotoxic than AuNCs-S (42% cell death). Interestingly, AuNCs functionalized with both chemotherapeutic drugs (AuNCs-DS) exhibited enhanced cytotoxicity. Furthermore, the most active nanostructure (AuNCs-DS) was further assessed at three different times (24 h, 48 h, and 72 h) in three cell lines (MCF7, MDA-MB-231, and Panc-1), revealing similar results (Figure S4).

**Figure 5:**
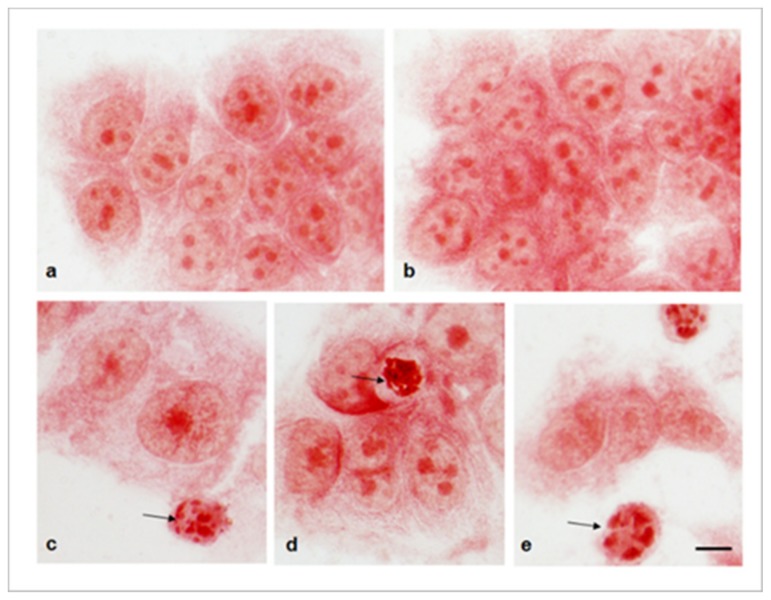
Morphology of MCF-7 cells stained with Neutral Red 48 h after treatment. (**a**) Control cells; (**b**–**e**) Cells incubated with AuNCs, AuNCs-D, AuNCs-S, and AuNCs-DS, respectively. Arrows indicate nuclei with apoptotic nuclear morphology (chromatin condensation and fragmentation). Scale bar: 10 μm.

The antitumoral activity of the nanostructures in MCF7 was confirmed in the morphological changes produced by the functionalized nanostructures during a neutral red staining assay. Particularly, cells incubated with AuNCs (Figure 5b) showed similar morphology to control cells (Figure 5a). On the contrary, samples incubated with AuNCs-D, or AuNCs-S and AuNCs-DS, showed shrunken cells with condensed or fragmented apoptotic nuclei (Figure 5c–e, respectively).

**Figure 6:**
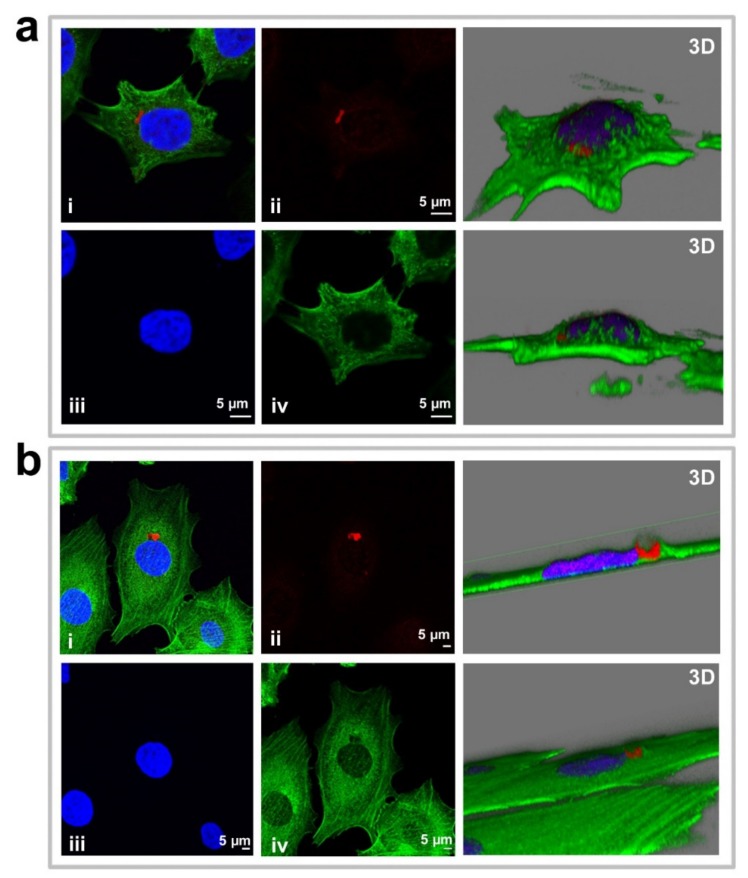
Confocal laser scanning microscope images of AuNCs localization in MCF7 cells after 3–4 h of incubation. Panel **a** shows an example of AuNCs cellular internalization while panel **b** shows AuNCs layered outside, on top of the cells. The left side of the panel corresponds to 2D images (one section or focal plane) for the merge (i), AuNCs in red (ii, Ex.405/Em.680 nm), nucleus with DAPI in blue (iii, Ex.358/Em.461) and actin filaments labeled with Phalloidin in green (iv, Ex.495/Em.519 nm) while the right side shows the 3D reconstructions of those cells.

The advantage of the bifunctional nanostructure was confirmed by studying cell damage using an inverted microscope. Particularly, the cell density observed when AuNCs-DS was employed (Figure S5d) was lower compared to the other two formulations (Figure S5b,c) and the control (Figure S5a). However, the most prominent change was observed after 9 days, when cells pre-treated with AuNCs-DS did not regrow, and the characteristic microcolonies of MCF-7 cells were no detectable (Figure S5g). On the other hand, when the cells were pre-incubated with AuNCs-D (Figure S5e) or AuNCs-S (Figure S5f), small microcolonies could be visualized, evidencing the superior activity of the bi-functionalized AuNCs. Then, we confirmed that AuNCs were internalized into MCF-7 cells by ICP-MS and confocal microscopy. Particularly, at short times (3–4 h after treatment), AuNCs are clearly detected through its intrinsic fluorescence inside the cells (Figure 6a) and outside the cell membrane (Figure 6b). At longer times (24 h post-treatment), AuNCs fluorescence is sparse, probably due to the expected degradation of the nanoparticle inside the cell, although the presence of Au could be detected by ICP-MS (13 pg/cell). In this period, a substantial fraction of the red fluorescence from DOX co-localizes with blue SN-38 (Figure S6). These results showed that SN38 and DOX can reach the cell nucleus after their release from the nanoparticle, leading to the corresponding cytotoxic effect on the cells.

**Figure 7:**
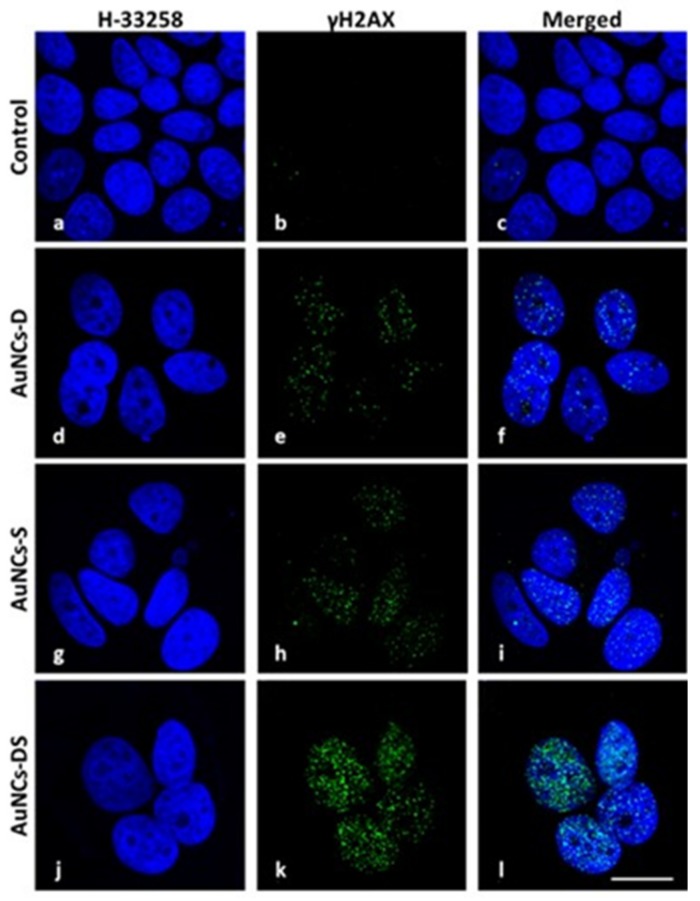
DNA damage study against histone γH2AX. γH2AX fluorescence pattern (green) was observed at the confocal microscope at 48 h after incubation with different AuNCs. MCF-7 cells were fixed and processed for γH2AX immunofluorescence, DNA was counterstained with Hoechst-33258 (blue) and both images were overlapped. (**a**–**c**) Control cells. (**d**–**f**) Cells pre-incubated with AuNCs-D. (**g**–**i**) Cells pre-incubated with AuNCs-S. (**j**–**l**) Cells pre-incubated with AuNCs-DS. Scale bar: 7.5 μm.

We further studied the effect of the conjugates through immunofluorescence. Particularly, we assessed their effect on the generation of DNA breaks using antibodies against histone H2AX at serine 139 (γ-H2AX). The fluorescent intensity was higher when the two chemotherapeutics were combined (Figure 7 and Figure S7).

### 2.3. Chemotherapeutic Activity of Functionalized AuNCs in MCF7 Mammospheres

**Figure 8:**
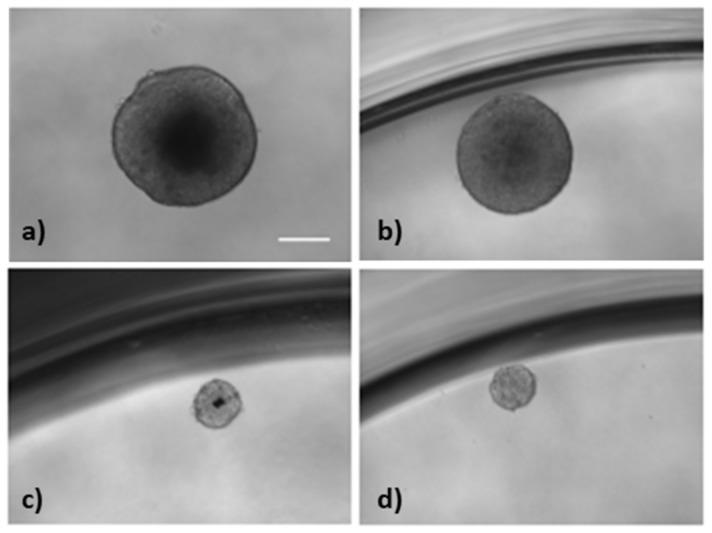
Activity of AuNCs in MCF7 mammospheres. (**a**) Control; (**b**) treated with AuNCs, (**c**) AuNCs-DS-(1), and (**d**) AuNCs-DS-(2), respectively. (1) means 0.08 µM concentration of the bifunctional complex, whereas (2) reflects treatments with the nanoconjugate at 0.6 µM. Scale bar 200 µm.

Finally, we evaluated the activity of the bifunctional complex in mammospheres using two concentrations, 0.08 µM (1) and 0.6 µM (2) based on preliminary optimization experiments done with mammospheres. The nanostructure was able to reduce the size of the mammospheres, from 465 µm (control) to 176 and 167 µm (Figure 8 and Figure S8).

**Figure 9:**
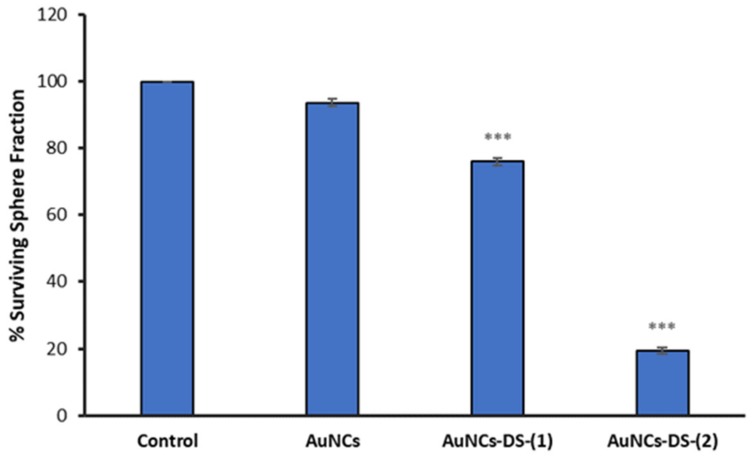
Surviving fraction of mammospheres treated with AuNCs for 7 days. Data correspond to mean ± S.D. values from three experiments. Statistical analysis was performed using one-way ANOVA Tukey’s test (each group vs Control). *** *p* < 0.0001.

The surviving fraction of mammospheres was also analyzed by counting the spheres before and after the treatment, and the results show a 24% and 81% reduction when treated at 0.08 µM and 0.6 µM, respectively (Figure 9).

## 3. Discussion

Breast cancer is the most frequent malignant tumor among women all over the world, except for non-melanoma skin cancer tumors. They are categorized according to different markers such as histopathology, tumor stage, grade, and receptor/gene expression [28,29,30,31,32]. To assess the potential of our approach to treat this disease, we have focused on two different breast cancer cells: MDA-MB-231 and MCF7. Even though both lines lead to the same kind of tumor, MDA-MB-231 is the “basal” type and triple negative (estrogen receptor (ER), progesterone receptor (PR), and human epidermal receptor 2 (HER2) negative) while MCF7 is “luminal” type and ER and PR positive. Such differences have an effect on their drug sensitivity. For instance, MCF7 presents a higher sensitivity to SN38 and Doxorubicin (DOX) (IC50 0.00790 µM and 0.00984 µM, respectively) compared to MDA-MB-231 (IC50 0.00978 µM and 1.24 µM, respectively) [33]. Those substantial sensitivity differences, along with the distinct clinical prognosis of both cell lines derived tumors, encourage us to choose them as good candidates for testing our therapeutic system. Moreover, in the case of MCF7, we were able to compare the standard 2D-adherent cell cultures to their corresponding mammospheres, which are 3D discrete spherical-shape clusters of cells enriched in CSCs [34].

Furthermore, we tested our system in PANC-1 cell line, which is a well-established model for Pancreatic Ductal Adenocarcinoma (PDAC). PDAC is an orphan disease with a bad prognosis, even when diagnosed early, and its survival rate after 5 years (<5%) has not changed over the last 30 years. In this work, we tested for the first time, to our knowledge, the combination of SN38 and DOX conjugated to AuNCs as a potential therapy against Pancreatic cancer.

The required multifunctional nanostructure was obtained using tailor-made linkers that facilitate the conjugation of the drugs and control their release. Due to the differences in hydrophilicity between SN38 (hydrophobic) and DOX (hydrophilic), their co-delivery using one vehicle is not straightforward [35,36]. In our case, we managed to conjugate DOX and SN38 onto the surface of AuNCs using tailored linkers, which can release the drugs upon specific cell internal stimuli (Figure 1) [37,38]. Particularly, we have exploited the low pH present in the endosomes (pH 5–6) or lysosomes (pH 4–5) [39,40] to trigger the release of DOX. The designed linker [41] contains a pH sensitive imine moiety to control the release of DOX, and a maleimide group to ease its conjugation with the previously sulfhydryl-activated AuNCs. The introduction of thiol moieties on the surface of AuNCs is easily carried out in one step and provides a convenient way to modify the structure with the drugs further. On the other hand, the high concentration of glutathione (1–10 mM) in the cytoplasm provides the required reducing environment to trigger the release SN38 [42,43]. Here, the linker employed contains a disulfide-based self-immolative moiety and a 2-mercaptopyridyl leaving group to ease the conjugation with the sulfhydryl-activated AuNCs [44]. The conjugation of one drug (AuNCs-D and AuNCs-S) or two (AuNCs-DS) onto AuNCs using these linkers was achieved just by mixing the components in a single step. Hence, this methodology provides an easy and efficient way of preparing multidrug delivery systems. Since the chemical transformations employed in developing this system are robust and widely used, integrating other drugs should be possible, enabling the exploration of other chemotherapeutic combinations. Also, the linkers employed allow the release of the drugs with no modification, ensuring that they reach their corresponding targets in their most active form. Using different linkers on the same nanostructure for the controlled release of therapeutic agents is scarce and to the best of our knowledge has not been employed to prepare BSA-stabilized AuNCs.

Interestingly, the conjugation of the drugs onto AuNCs induced a rapid formation of nanoparticles due to the self-assembly of drug loaded AuNCs [45]. This rearrangement is driven by the interactions between hydrophobic BSA domains, and the SN38 [46]. Dynamic light scattering measurements of the AuNCs-D revealed the formation of a non-homogeneous material with two nanoparticle size distributions (Figure 2), while the use of the hydrophobic SN38 monodispersed nanoparticles were obtained. The combined use of DOX and SN38 does not disrupt the formation of monodispersed nanoparticles, which were surprisingly stable in PBS for at least 15 days. SEM micrographs showed globular structures as found in other albumin-based nanoparticles (Figure S2) [47]. It is noteworthy that albumin-stabilized AuNCs based nanoparticles could accumulate better in tumor tissues due to their higher size compared with AuNCs (≤10 nm), since blood vessels of tumor tissues are larger than 10 nm [48].

Since DOX and SN38 were conjugated using respective sensitive linkers to pH and reductive environment, the drug release was investigated using different conditions (Figure 3). The DOX was efficiently released when the pH was reduced to 5 using a citrate-based buffer and SN38 when exposed at 1 mM of dithiothreitol (DTT).

The antitumoral effect of the nanostructures was assessed in MCF7 cells, where the three systems showed antitumoral activity. The best inhibition was obtained with the construction that carries the two chemotherapeutics (AuNCs-DS) (Figure 4). This combined system was also tested in additional breast cancer (MDA-MB-231) and pancreatic cancer (Panc-1) cell lines at different times after treatment (24 h, 48 h, and 72 h), showing similar results and highlighting the potential of the approach and its efficacy compared with the results obtained with the free drugs (Figure S4).

These results are in agreement with previous studies, where DOX and SN38 induced senescence, mitotic catastrophe, apoptosis, or necrosis, depending on the dose of the drugs [20,49]. In our case, MCF-7 cells undergoing apoptosis after treatment with mono- and bi-functional AuNCs.

It is known that DOX and SN38 are inducers of DNA damage and activate the DNA damage response (DDR) [50]. For this reason, we wonder if the superior activity of AuCNs-DS was related to the DNA damage generated by the structures. In this regard, it is well known that the phosphorylation of histone H2AX at serine 139 (γ-H2AX) is the most sensitive marker to examine DNA damage (double-stranded breaks, DSBs) in cells exposed to ionizing radiation or DNA-damaging chemotherapeutic agents. These phosphorylation events are easily detected as nuclear foci by specific antibodies to the phosphorylated form of H2AX (γ-H2AX), and the foci formation has been extensively used as a marker of DSB formation [51].

Since mono- or bi-functionalized gold nanoclusters need to be internalized by cells and, subsequently, chemotherapeutic drugs must enter to cell nuclei to induce cell damage, we performed an analysis of DNA double-strand breaks in MCF-7 cells to detect phosphorylation of H2AX by immunofluorescence techniques following the treatment with different loaded AuNCs. As seen in Figure 7, γ-H2AX fluorescence signals were detected in both, AuNCs-D and AuNCs-S, although the fluorescent intensity in cells treated with bi-functionalized AuNCs was significantly higher. This result suggests that the enhanced activity observed in the bifunctional nanostructures might be due to increased DNA damage generated by the combination of the two drugs at the nuclei. In addition, optical microscopy studies revealed that the AuNCs-DS induces higher morphological damage after 48 h and prevents the generation of colonies after 9 days, compared to the other formulations (Figures S7 and S8).

To further assess the therapeutic potential of this nanostructure, we decided to test it against mammospheres. These spherical structures resemble better the tumor environment, compared with standard 2D cell culture [52], and what is more, is an excellent model to test therapeutics against cancer stem cells (CSCs). CSCs are small cell populations with self-renewing and high tumorigenic capabilities, which is responsible for drug resistance and relapses [53,54]. This kind of cell is present in a vast variety of tumors [55,56] such as glioblastomas [57] or breast cancer [58].

Standard chemotherapeutics do not remove this subpopulation of a tumor, due to their inherent drug resistance and its efficient DNA repair system [59] For these reasons, we believe that our nanoconjugated system might be useful to control this CSCs population due to its inherent efficiency in the generation of DNA damage, as shown in Figure 7 and Figure S7.

We assessed the activity of AuNCs-DS at different concentrations in MCF7 cells. We selected two concentrations 0.08 µM (1) and 0.6 µM (2) for the mammosphere experiments based on this data and preliminary experiments with mammospheres. The high potency of the conjugates obliged us to use these concentrations in the mammosphere assay model to prevent the complete elimination of the spheres after 7 days of treatment. At the selected concentrations, we observed a significant variation on the size of the mammospheres, which are reduced from an average diameter of 465 µm (control) to 176 and 167 µm (Figure 8 and Figure S8).

We also analysed the surviving fraction of mammospheres by counting the spheres before and after the treatment, and the results show a 24% and 81% reduction when treated at 0.08 µM and 0.6 µM, respectively (Figure 8).

## 4. Materials and Methods

### 4.1. Synthesis of AuNCs

To a solution of BSA (1 mL, 50 mg/mL) at 37 °C, hydrogen tetrachloroaurate (III) hydrate (HAuCl_4_) (1 mL, 10 mM) was added at 37 °C and stirred for 2 min. Then, 100 μL of NaOH 1M was added, and the mixture was stirred for 24 h. AuNCs were purified using an exclusion column NAP-10 of Sephadex-G25 according to the specifications of the distributor.

### 4.2. Sulfhydryl Activation of AuNCs

To a solution of AuNCs (1 mL, 20 μM) in PBS (pH = 7.8), a solution 6.5 mM of 2-iminothiolane hydrochloride (77 µL) was added and incubated at room temperature for 16 h. The product was purified using an exclusion column NAP-10 of Sephadex-G25 according to the specifications of the distributor.

### 4.3. Functionalization of AuNCs

To synthesize AuNCs-D, 100 μL of a solution of modified DOX (3) (1 mM) in DMF was added to 1 mL of a solution of sulfhydryl-activated AuNCs (20 μM) in PBS (pH = 7.8) and stirred at room temperature for 16 h. Then, AuNCs-D were purified using a NAP-10 column. AuNCs-S were prepared as above by using 150 μL of a solution of modified SN38 (6) (1 mM) in DMF. The synthesis of AuNCs-DS was carried out using the same procedure by adding 100 μL of a solution of modified DOX (3) (1 mM) in DMF, immediately followed by the addition of 100 μL of a solution of modified SN38 (6) (1 mM) in DMF.

### 4.4. Quantification of Drug Functionalization

The drug functionalization was determined using UV-Vis spectroscopy. In brief, the absorption of DOX (490 nm) and SN38 (380 nm) of the corresponding formulations were measured, and the concentration quantified by interpolation from a calibration curve.

Drug loading was calculated by following the formula:

$$\mathrm{Drug}\;\mathrm{Loading}\left(\mathrm{weight}\;\%\right)=\frac{\mathrm{weight}\;\mathrm{of}\;\mathrm{drug}\;\mathrm{in}\;\mathrm{nanoparticles}}{\mathrm{total}\;\mathrm{weight}\;\mathrm{of}\;\mathrm{nanoparticles}}\times 100$$

### 4.5. Dynamic Light Scattering Measurements

Dynamic light scattering (DLS) measurements were performed at 25 °C at a 173° scattering angle using disposable microcuvettes. The Z-Average hydrodynamic diameter and polydispersity index (PDI) were obtained by three cumulative analyses of 100 μL of the corresponding formulation of AuNCs (20 μM).

### 4.6. Scanning Electron Microscopy

One drop of a solution of corresponding functionalize AuNCs was deposited over a silica wafer and air-dried during 16 h. Then, the samples were observed using a Carl Zeiss AURIGA scanning electron microscope (Zeiss, Jana, Germany).

### 4.7. In Vitro Doxorubicin (DOX)/SN38 Release

DOX release profile was evaluated using fluorescence spectrophotometry. 1 mL of a solution of DOX-AuNCs in saline citrate buffer at pH = 7 or 5 were incubated at 37 °C. At different time intervals, 100 μL of this solution were withdrawn and treated with 100 μL of a 2% ZnSO4 solution in H20/MeOH (1:1). After vigorous stirring, this mixture was centrifuged at 13200 rpm for 10 min, and the fluorescence of the DOX released analyzed by fluorescence from the supernatant (ʎ_exc_ = 495 nm, ʎ_em_ = 590 nm). SN38 release profile was evaluated using a similar protocol. In this case, 1 mL of a solution of SN38-AuNCs in PBS at pH = 7.4 containing dithiothreitol at 1 µM or 1 mM of concentration was incubated, and the SN38 released analyzed by fluorescence from the supernatant (ʎ_exc_ = 370 nm, ʎ_em_ = 550 nm).

### 4.8. Cell Culture

Human breast adenocarcinoma (MCF-7 and MDA-MB-231) and human pancreatic adenocarcinoma (Panc-1) cells were obtained from American Type Culture Collection (ATCC)^®^. Both cell lines were grown as monolayer cultures in Dulbecco’s modified Eagle’s medium (DMEM) supplemented with 10% (*v*/*v*) fetal bovine serum (FBS), 1% L-Glutamine, 50  U/mL penicillin, and 50 μg/mL streptomycin and incubated at standard conditions (37 °C, 5% CO_2_).

### 4.9. Neutral Red Staining

MCF-7 cells grown on coverslips in 24-well plates and incubated for 24 h with the nanostructures were fixed in cold methanol for 5 min and then stained with 0.5% neutral red for 2 min. Coverslips were washed with distilled water, air-dried, mounted in DePeX and examined by light microscopy.

### 4.10. Cytotoxicity Assays

#### 4.10.1. MTT Tetrazolium Reduction Assay

Cells were incubated with the nanostructures (AuNCs concentration of 2.6 μM) in complete cell culture media for 24 h. After incubation, the culture medium was removed, and samples were washed three times with phosphate-buffered saline (PBS, pH 7.4) and cells were incubated 48 h after treatment. Then, toxicity was assessed by MTT (dimethylthiazolyl-diphenyl-tetrazolium bromide) colorimetric assay. Briefly, the culture medium was replaced with DMEM containing MTT (5 mg/mL). Five hundred microliters of this MTT solution (50 μg/mL MTT in culture medium) was added to each culture dish and cells were incubated for 4 h at 37 °C. Then, MTT was removed by aspiration and reduced formazan crystals were dissolved with 500 μL of dimethylsulfoxide and the absorbance measured at 540 nm using a microplate reader. Cell survival was expressed as the percentage of absorption of treated cells in comparison with control cells. Besides, experiments with AuNCs non-functionalized were also performed, to exclude a possible cytotoxic effect exercised by gold nanoclusters per se. Data corresponded to mean values ± standard deviation from at least five different experiments.

#### 4.10.2. Resazurin Reduction Assay

Breast Cancer (MCF7 and MDA-MB-231) and pancreatic cancer (Panc-1) cell lines were seeded (40,000 cells/well, 24 h prior treatment) in 24 well plates and incubated in standard conditions. Then, the cells were incubated with the nanostructures using a constant concentration of AuNCs (2.6 µM) or the free drugs (1.1 µM DOX or 2.4 µM SN38) for 24 h. After incubation, the culture medium was removed, and cells were washed twice with PBS (pH = 7.4). Toxicity was measured 24, 48, and 72 h after treatment, using the resazurin assay following the manufacturer’s protocol. Briefly, the cell culture medium was replaced with fresh culture medium containing 1% of resazurin solution (1 mg/mL in PBS), and cells were incubated for 3 h more at 37 °C, 5% CO_2_. Fluorescence from 100 µL of the culture media was measured using a microplate reader ((λexc = 550 nm, λem = 590 nm). Cell viability was expressed as a percentage of fluorescence of treated cells and compared with the control. Data corresponded to mean values ± standard deviation from at least three different experiments 

### 4.11. Inductively Coupled Plasma Mass Spectrometry (ICP-MS)

MCF7 cells treated with 2.3 µM AuNCs for 24 h were harvested and quantified, prior digestion O/N in aqua regia and subsequent dilution in pure water. After that, the quantity of Au^+^ in the sample was determined by ICP-MS and relativized per cell unit.

### 4.12. Live Cell Imaging

Cells were incubated with the nanostructures for 48 h, washed three times with PBS, and maintained in culture medium for 9 days, and the culture medium was changed every 2–3 days. Untreated control, as well as cells incubated with AuNCs without a drug, were also visualized. Cells were imaged daily under a differential interference contrast (DIC) inverted microscope (Leica DMI 6000B) equipped with a Leica DFC420 C digital camera (Leica Microsystems, Heerbrugg, Switzerland).

### 4.13. Subcellular Localization

#### 4.13.1. AuNCs at Short Times (3–4 h after Treatment)

MCF7 cells grown in 24 well plates with glass coverslips were treated with 50 µM AuNCs during 3–4 h and fixed afterward with 4% paraformaldehyde for 15 min/RT. Fixed cells were subsequently permeabilized and labeled for 20 minutes/RT in darkness with a mix containing Saponin 0.25% (Sigma, Saint Louis, MO, USA), DAPI (diluted 1:300 from a 1 mg/mL stock solution, Sigma), Phalloidin (diluted 1:250 from a 1 mg/mL stock solution, Sigma), and FBS 5% in PBS buffer. After washing 3 times with PBS, coverslips were mounted onto slides using Fluoroshield medium (Sigma) and visualized by confocal microscopy (Confocal multispectral Leica TCS SP8 system). Data acquisition was performed with Leica software LAS X and images were prepared with ImageJ (https://imagej.nih.gov/ij/). To avoid possible artifacts due to the crosstalk between the red and the blue channels, the blue signal was subtracted from the red 2D images using ImageJ processing.

#### 4.13.2. AuNCs-DS at Long Times (24 h after Treatment)

Internalization of bifunctionalized AuNCs into MCF-7 cells were visualized by confocal microscopy. Cells grown on coverslips were incubated for 24 h with AuNCs-DS, washed three times with culture medium without FBS, and visualized under differential interference contrast (DIC) microscopy and confocal fluorescence microscopy using a multispectral Leica TCS SP5 confocal microscope, operating with 405 Diode (UV) and DPSS (561, visible) laser lines.

### 4.14. Microscopic Detection of DNA Damage

MCF-7 cells grown on glass coverslips and incubated with the different AuNCs for 24 h were immunostained for histone phosphorylated H2AX (γ-H2AX) at 48 h after treatments. Cells were fixed with formaldehyde in PBS (1:10 *v*/*v*) for 20 min, washed three times for 5 min with PBS, and then permeabilized with 0.5% Triton X-100 in PBS for 5 min. After incubation with a blocking solution (5% bovine serum albumin, 5% FBS, 0.02% Triton X-100 in PBS) at room temperature for 30 min, cells were washed three times with PBS. Then, cells were incubated with primary monoclonal mouse anti-γ-H2AX antibody (Merck Millipore, Darmstadt, Germany) diluted 1:100 at 37 °C in a wet chamber for 1 h. After three washes with PBS, incubation with secondary antibody (Alexa Fluor^®^ 488 goat anti-mouse, Life Technologies (Waltham, MA, USA) was identical to that of the first one and so were final washings. Finally, DNA was counterstained by addition of Hoechst-33258 (0.05 mg/mL in distilled water) for 5 min, and the sample was mounted with ProLong Gold antifade reagent. Immunofluorescence images were captured using a laser scanning confocal microscope using a multispectral Leica TCS SP5 confocal microscope (Leica, Wetzlar, Germany), operating with 405 nm (argon–UV) and 488 nm (argon) laser lines. All images were taken with a photomultiplier value (PTM) of 1029.

### 4.15. Mammosphere Culture

Single-cell suspensions of MCF-7 cell line were plated in P96 well plates at 10 cells per well in DMEM/F-12 medium supplemented with GlutaMAX, B27 (Gibco: Thermo Fisher, Waltham, MA, USA), 10 ng/mL epidermal growth factor (EGF: Invitrogen) and 10 ng/mL basic fibroblast growth factor (bFGF: Millipore, Burlington, MA, USA) and they were maintained at 37 °C in 5% CO_2_. At day 10, the number of wells with mammospheres were counted and then nanostructures were added. The final proportion of living spheres were quantified at day 17.

### 4.16. Statistical Analysis

For statistical calculations, one-way ANOVA Tukey’s test and the R (R Foundation for Statistical Computing, Vienna, Austria) were used. *p* values < 0.05 (*), <0.01 (**), and < 0.001 (***) were considered as statistically significant.

## 5. Conclusions

Toxicity and side effects are serious drawbacks that hinder the use of the chemotherapeutics to treat tumors. Furthermore, the development of drug resistance contributes to a great extent to the failure of current treatments. In this regard, the combination of two or more agents allows a reduction in the number of individual components, decreasing their toxicity and preventing the development of resistance by tumor cells. However, the rapid clearance and the low accumulation of the drugs in the tumor are drawbacks that must be solved. In this sense, nanomedicine tries to tackle these problems using nanostructures capable of delivering drugs more efficiently and safely.

In this work, we have demonstrated that bovine serum stabilized AuNCs can be used as safety carriers of two chemotherapeutics such as DOX and SN38. We have used tailored linkers to functionalize the AuNCs with the drugs, which render in the spontaneous formation of nanoparticles with a diameter below 200 nm. It is worth mentioning that the chemistry employed is robust and the system could be adapted to other drug combinations. Two internal cell stimulus controls the release of the drugs. In particular, DOX is released at low pHs, such as that found in the endosomes and lysosomes. The release of SN38 takes place in a reducing environment equivalent to the one present in the cytoplasm. AuNCs have not shown toxicity in MCF-7 cells. However, AuNCs-D and AuNCs-S evidenced a reduction in cancer cell viability. Remarkably, this activity was enhanced when we used bi-functional nanoparticles (AuNCs-DS). This particular cytotoxic activity might be of interest to reduce CSCs subpopulation, which is involved in metastasis and recurrences. The immunofluorescent studies carried out suggest that the superior activity of the bi-functional nanostructure might be due to the prominent DNA damage generated. Interestingly, our system is also active in the reduction of the size and number of mammospheres, a model of CSC.

## Supplementary Materials

The following are available online at https://www.mdpi.com/2072-6694/11/7/969/s1, Supplementary Materials and Methods: Synthesis of Intermediates and Pro-Drugs, Figure S1: UV-Vis spectra of AuNCs, and AuNCs modified with DOX (AuNCs-D), SN38 (AuNCs-S) and both DOX and SN38 (AuNCs-DS), Figure S2: SEM pictures of modified AuNCs. (a) AuNCs-D, (b) AuNCs-S and (c) AuNCs-DS, Figure S3: Stability study in PBS of AuNCs-DS over time stored at room temperature. Mean ± SD, *n* = 3, Figure S4: Surviving fraction of breast cancer MCF-7, MDA-MB-231 and pancreatic cancer Panc-1 cells incubated 24 h with AuNCs and AuNCs-DS or the free unmodified drugs and evaluated at different times a) 24 h; b) 48 h; and c, d) 72 h after treatment by resazurin viability assay, Figure S5: MCF-7 cell density recorded under Differential Interference Contrast (DIC) microscopy 48 h after incubation in control (untreated) cells (a) or incubated with AuNCs-D (b), AuNCs-S (c) and AuNCs-DS (d), Cells 9 days after incubation with AuNCs-D (e), AuNCs-S (f) and AuNCs-DS (g), Figure S6: Confocal laser scanning microscope images of subcellular localization of AuNCs-DS after 24 h incubation, Figure S7: Foci detected by γH2AX immunofluorescence analysed with ImageJ software, Figure S8: Size of mammospheres treated with AuNCs-DS at 0.08 µM (1) and 0.6 µM (2) for 7 days.
