# Supplementary material for: Multifunctional Albumin-Stabilized Gold Nanoclusters for the Reduction of Cancer Stem Cells
Source: Cancers (Basel). 2019 Jul 10;11(7):969. doi: 10.3390/cancers11070969 (PMC6678462; doi:10.3390/cancers11070969)
Supplement: Supplementary file 1 [file cancers-11-00969-s001.pdf]

Electronic Supporting Information (ESI)
Multifunctional Albumin-Stabilised Gold Nanoclusters for the Reduction of Cancer Stem Cells

Ana Latorre, Alfonso Latorre, Milagros Castellanos, Ciro Rodriguez Diaz, Ana Lazaro-Carrillo, Tania Aguado, Mercedes Lecea, Sonia Romero-Pérez, Macarena Calero, José María Sanchez-Puelles, Ángeles Villanueva and Álvaro Somoza

Synthesis of Intermediates and Pro-Drugs

Materials and methods: ^{1}H and ^{13}C NMR spectra of intermediates were recorded in $CDCl_3$ at 300 and 75MHz, respectively. All reactions were monitored by thin layer chromatography using precoated sheets of silica gel 60. Flash column chromatography was done using silica gel 60 (230-400 mesh, Merck). Eluting solvents are indicated in the text. Doxorubicin and SN38 were purchased from Fluorochem and $HAuCl_4$ from ABCR. All other reagents were purchased from Sigma-Aldrich and used without further purification.

1. Doxorubicin modified with a pH-sensitive linker

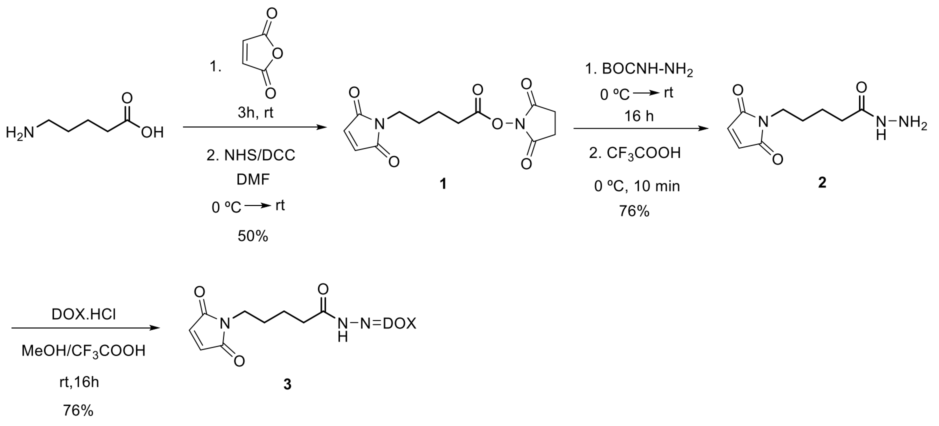

1.1. Succinimido 5-maleimidopentanoate (1)[1]

To a solution of maleic anhydride (167 mg, 1.7 mmol) in DMF (2 mL) under argon, aminovaleric acid (167 mg, 1.7 mmol) was added, and the mixture stirred at room temperature for 3h. Then, NHS (234 mg, 2 mmol) and DCC (700 mg, 3.4 mmol) were added at 0 °C. After 20 minutes, the mixture was warmed up to room temperature and stirred for 16 h. The resulting precipitate was filtered off, and the solvent concentrated in vacuo. The residue was purified by flash chromatography (eluent CH_2Cl_2/Isopropanol 97:3) to give the compound **1** in 50% yield as white solid; ^{1}H NMR (300 MHz, $CDCl_3$) δ 6.69 (bs, 2H), 3.55 (bs, 2H), 2.82 (bs, 4H), 2.64 (bs, 2H), 1.72 (bs, 4H).

1.1 5-Maleimidovalerohydrazide trifluoroacetic acid salt (2)[2]

To a solution of **1** (30 mg, 0.1 mmol) in CH_2Cl_2 (1 mL), *tert*-butyl carbazate (15 mg, 0.1 mmol) was added at 0 °C. Then, the solution was let to reach room temperature slowly and stirred for 16 h. The solution was concentrated in vacuo, and the residue purified by flash chromatography (eluent CH_2Cl_2/MeOH 100:1 first and gradually changed to 30:1) to obtain the Boc-protected compound **2** as colorless oil; ^{1}H NMR (300 MHz, $CDCl_3$) δ 6.68 (bs, 2H), 3.52 (bs, 2H), 2.25 (bs, 2H), 1.63 (bs, 4H), 1.44 (s, 9H).

This intermediate was dissolved in 0.5 mL trifluoroacetic acid previously cooled at 0 °C, and stirred for 10 minutes. The solution was concentrated in vacuo and the resultant solid purified by washing with Et_2O to obtain compound **2** as dark white solid in 76% yield.

1.1. (5-Maleimidovaleroyl) hydrazine of Doxorubicin (3)[2]

To a solution of compound **2** (15 mg, 0.071 mmol) and Doxorubicin (13 mg, 0.023 mmol) in MeOH (4.5 mL), two drops of trifluoroacetic acid were added and stirred at room temperature for 16 h. Then, the solution was concentrated in vacuo, and the residue dissolved in the minimal amount of MeOH. To this solution, CH_3CN was added, and the mixture cooled to 4 °C during 16 h. The solvent was decanted and the solid washed with CH_2Cl_2 to obtain compound **3** as red solid in 76 % yield; ^1H NMR (300 MHz, MeOD) δ 7.87-7.80 (m, 2H), 7.54 (d, 1H), 6.76 (s, 2H), 5.48 (s, 1H), 5.07 (s, 1H), 4.74-4.64 (m, 2H), 4.26-4.21 (m, 1H), 4.02 (s, 3H), 3.70-3.54(m, 3H), 3.41 (s, 2) 2.59-2.29 (m, 3H), 2.11-1.88 (m, 2H), 1.67-1.53 (m, 5H), 1.32 (t, 3H). MS (ESI): *m/z* (%) 737 (M^++H, 100), 759 (M^++Na, 2); HRMS (ESI) calcd for $C_{36}H_{41}N_4O_{13}$ (M^++H) 737.2638, found 737.2664.

1. SN38 modified with a redox-sensitive linker

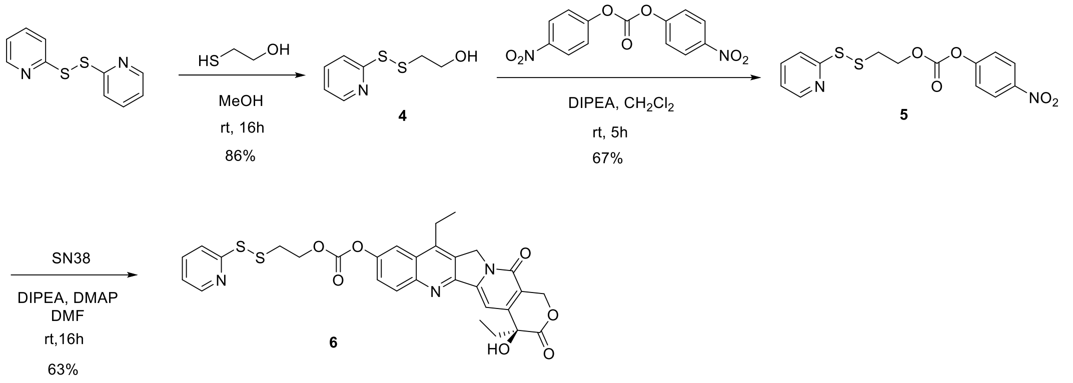

2.1 2-(Pyridin-2-yldisulfanyl)ethanol (4)[3]*:*

To a solution of aldrithiol (300 mg, 1.36 mmol) in MeOH (1.5 mL) under Ar, 2-mercaptoethanol (53 µL, 0.75 mmol) was added slowly and stirred for 16 h. Then, the solvent was evaporated in vacuo and the residue purified by flash chromatography (eluent CH_2Cl_2/AcOEt 5:1) to obtain compound **4** as a colorless oil in 86 % yield; ^1H NMR (300 MHz, $CDCl_3$) δ 8.51 (d, J = 4.3 Hz, 1H), 7.58 (td, J = 8.0, 1.7 Hz, 1H), 7.40 (d, J = 8.0 Hz, 1H), 7.17–7.13 (m, 1H), 5.72 (bs, 1H), 3.80 (dd, J = 10.4, 6.5 Hz, 2H), 2.97–2.94 (m, 2H).

2.1 4-Nitrophenyl 2-(pyridin-2-yldisulfanyl)ethyl carbonate (5)[3]*:*

To a solution of compound **4** (100 mg, 0.53 mmol), and bis(4-nitrophenyl) carbonate (241 mg, 0.79 mmol) in CH_2Cl_2 (2 mL) under Ar, DIPEA (158 µL, 0.79 mmol) was added and stirred for 5 h. The mixture was washed with water, and the organic phase dried with $MgSO_4$. After solvent evaporation, the residue was purified by flash chromatography (eluent Hexane/AcOEt 4:1 and then 2:1) to obtain compound **5** (Figure 1) as a colorless oil 67 % yield; ^1H NMR (300 MHz, $CDCl_3$) δ 8.50 (d, J = 4.8 Hz, 1H), 8.28 (d, J = 9.1 Hz, 2H), 7.72–7.59 (m, 2H), 7.38 (d, J = 9.1 Hz, 2H), 7.15–7.10 (m, 1H), 4.57 (t, J = 6.4 Hz, 2H), 3.16 (t, J = 6.4 Hz, 2H).

2.1 4-Nitrophenyl 2-(pyridin-2-yldisulfanyl)ethyl carbonate of SN38 (6)[4]*:*

To a solution of **5** and SN38 in DMF under argon, DIPEA and catalytic amount of DMAP were added and stirred during 16 h. Then, the mixture was concentrated in vacuo and the residue purified by flash chromatography (eluent CH_2Cl_2/MeOH 30:1) to obtain compound **6** as colorless oil in 63 % yield; ^1H NMR (500 MHz, $CDCl_3$) δ 8.42 (d, J = 4.8, 1.3 Hz, 1 H), 8.07 (d, J = 9.1 Hz, 1H), 7.66 (dd, J = 4.6, 1.3 Hz, 1H), 7.45 (dd, J = 9.1, 2.6 Hz, 1H), 7.36 (d, J = 2.6 HZ, 1H), 7.26 (s, 1H), 7.06 (h, J = 4.6 Hz, 1H), 5.71 and 5.40 (AB system, J = 17 Hz, 2H), 5.09 (bs, 2H), 4.39-4.29 (m, 2H), 3.05 (t, J = 6.6 Hz, 3H), 3.01-2.98 (m, 2H), 2.26 (dq, J = 14.8, 7.4 Hz, 1H), 2.14 (dq, J = 14.8, 7.4 Hz, 1H), 1.30 (t, J = 7.7 Hz, 3H), 0.99 (t, J = 7.5 Hz, 3H); ^{13}C NMR (500 MHz, $CDCl_3$) δ 167.4, 159.3, 157.2, 156.1, 153.5, 149.6, 149.0, 147.4, 145.9, 144.7, 143.6, 137.5, 132.0, 128.5, 126.9, 122.7, 121.1, 120.0, 119.3, 105.5, 95.5, 78.2, 67.1, 66.4, 49.4,

36.9, 31.2, 23.1, 13.6, 7.6; MS (ESI): *m*/*z* (%) 303 (20), 606 (M^++H, 100), 628 (M^++Na, 1); HRMS (ESI) calcd for $C_{30}H_{28}N_3O_7S_2$ (M^++H) 606.1377, found 606.1363.

Supplementary Figures and Tables

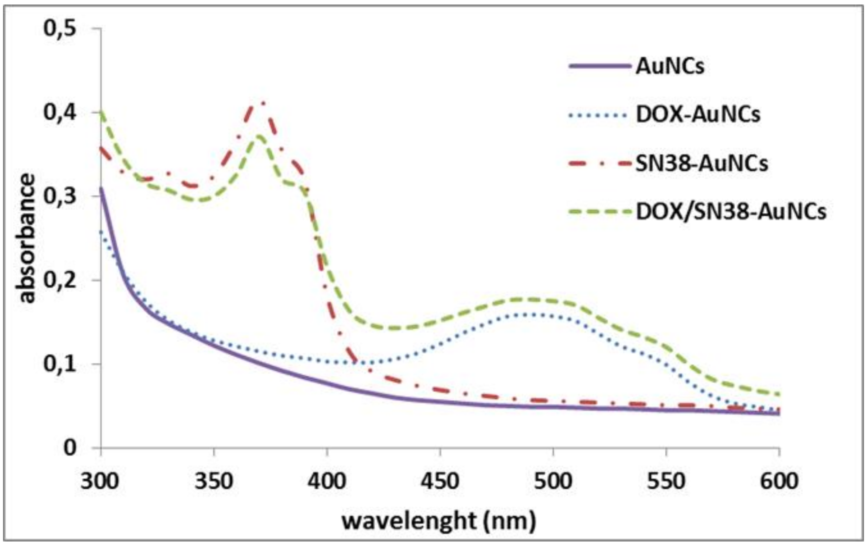

Figure S1. UV-Vis spectra of AuNCs, and AuNCs modified with DOX (AuNCs-D), SN38 (AuNCs-S) and both DOX and SN38 (AuNCs-DS).

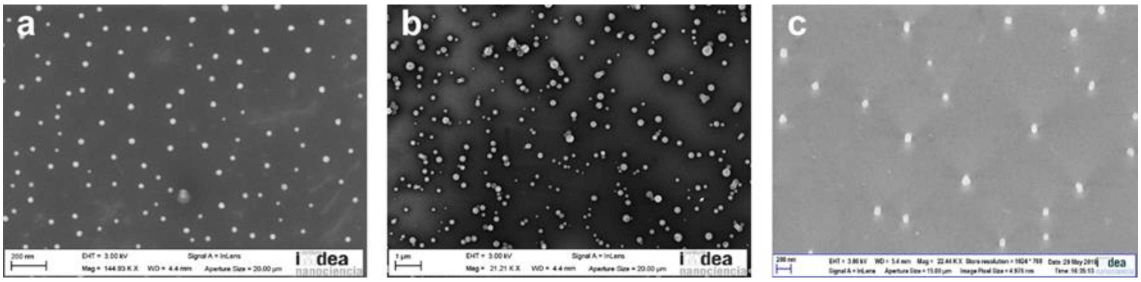

Figure S2. SEM pictures of modified AuNCs. (**a**) AuNCs-D, (**b**) AuNCs-S and (**c**) AuNCs-DS.

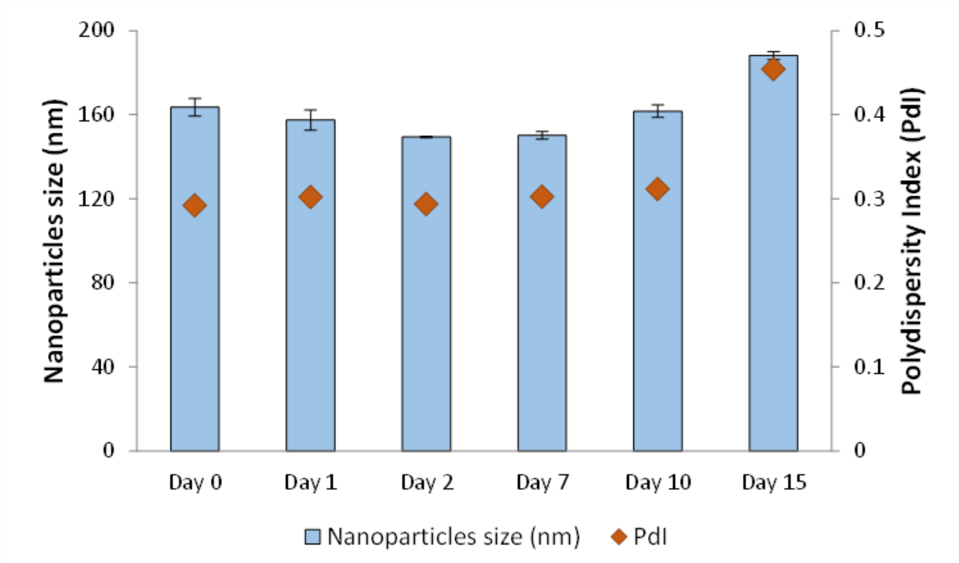

Figure S3. Stability study in PBS of AuNCs-DS over time stored at room temperature. Mean ± SD, *n* = 3.

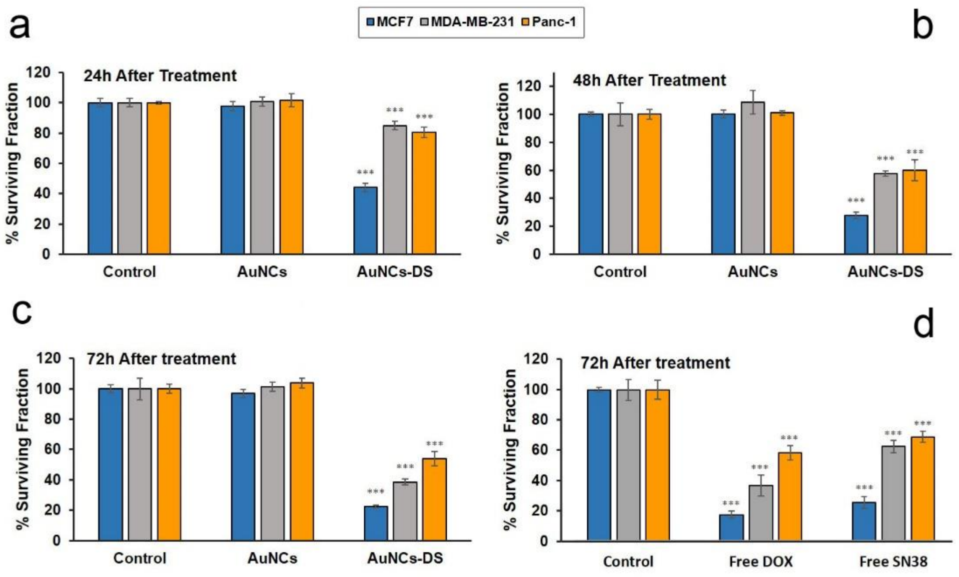

Figure S4. Surviving fraction of breast cancer MCF-7, MDA-MB-231 and pancreatic cancer Panc-1 cells incubated 24 h with AuNCs and AuNCs-DS or the free unmodified drugs and evaluated at different times (**a**) 24 h; (**b**) 48h; and (**c**,**d**) 72 h after treatment by resazurin viability assay. Data correspond to mean ± S.D. ($n = 4$). Statistical analysis was performed using one-way ANOVA Tukey's test (each group vs Control). *** $p < 0.0001$.

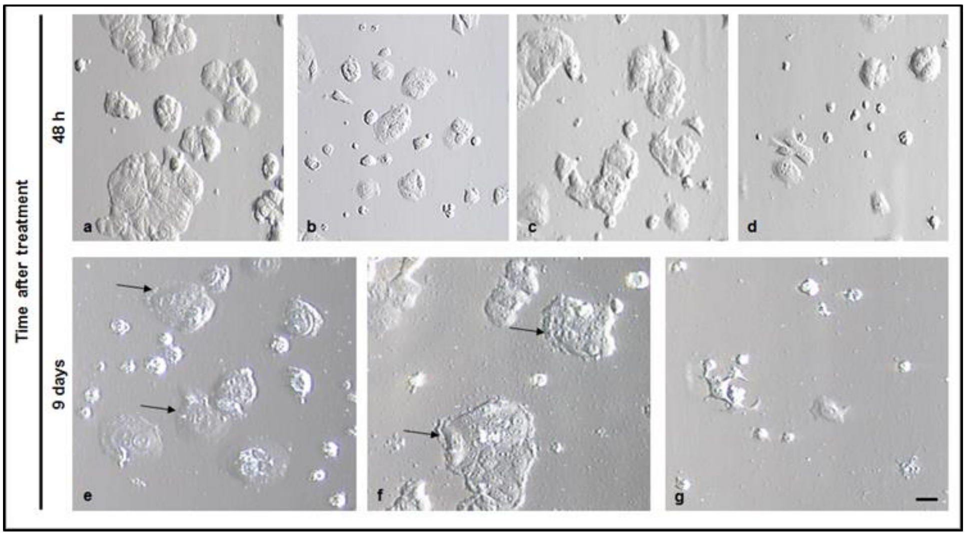

Figure 5. MCF-7 cell density recorded under Differential Interference Contrast (DIC) microscopy 48 h after incubation in control (untreated) cells (**a**) or incubated with AuNCs-D (**b**), AuNCs-S (**c**) and AuNCs-DS (**d**). Cells 9 days after incubation with AuNCs-D (**e**), AuNCs-S (**f**) and AuNCs-DS (**g**). Arrows indicate the growth of surviving cells, which forms new microcolonies. As detected under an inverted microscope, a fewer number of live cells was noted following treatment with bifunctional in contrast with the mono-functional AuNCs. Scale bar: 50 µm.

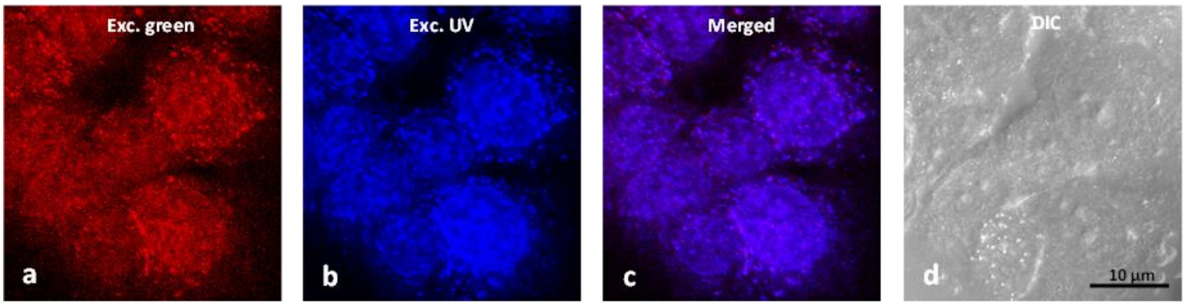

Figure S6. Confocal laser scanning microscope images of subcellular localization of AuNCs-DS after 24 h incubation. (**a**) DOX internalized are colored red; and (**b**) SN38 colored blue. (**c**) Merged images; and (**d**) differential interference contrast (DIC) microscopy are also presented. Scale bar 10 µm.

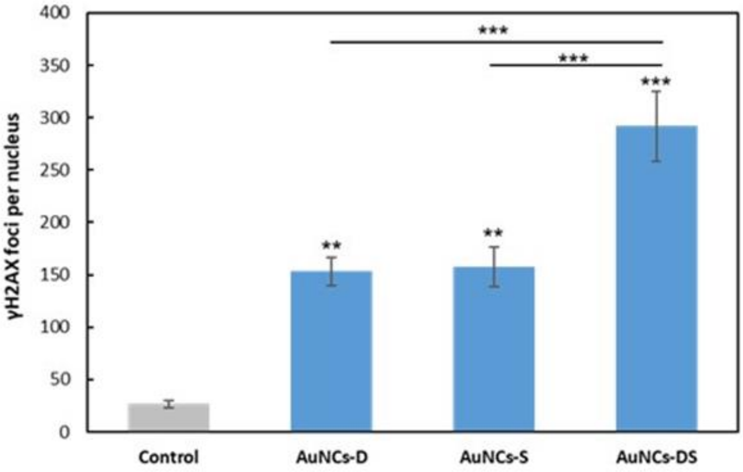

Figure S7. Foci detected by γH2AX immunofluorescence analysed with ImageJ software. Data correspond to mean ± SEM values. At least 50 nuclei were analysed. Statistical analysis was performed using one-way ANOVA Tukey's test. ** $p < 0.001$, and *** $p < 0.0001$.

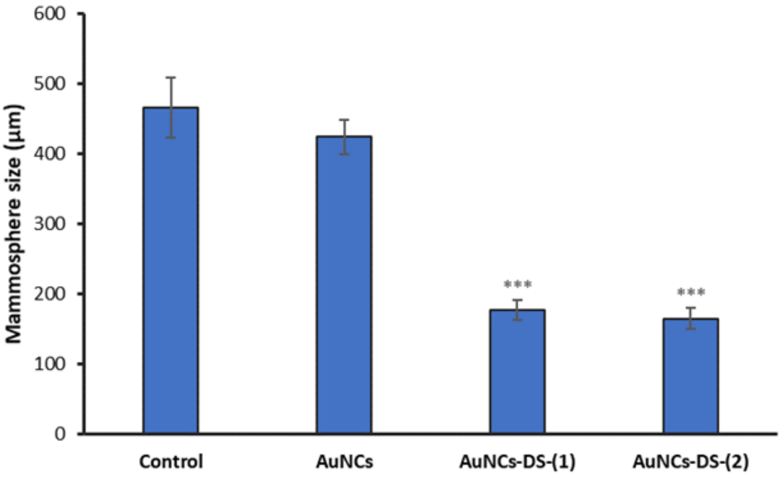

Figure S8. Size of mammospheres treated with AuNCs-DS at 0.08 µM (1) and 0.6 µM (2) for 7 days. Data correspond to mean ± S.D. values from ten mammospheres. Statistical analysis was performed using one-way ANOVA Tukey's test. *** $p < 0.0001$.

References

1. 1. Juárez-Hernández, R.E.; Miller, P.A.; Miller, M.J. Syntheses of Siderophore-Drug Conjugates Using a Convergent Thiol-Maleimide System. *ACS Med. Chem. Lett.* **2012**, *3* (10), 799–803.
2. 2. Willner, D.; Trail, P.A; Hofstead, S.J.; King, H.D.; Lasch, S.J.; Braslawsky, G. R.; Greenfield, R. S.; Kaneko, T.; Firestone, R.A. (6-Maleimidocaproyl)hydrazone of Doxorubicin. A New Derivative for the Preparation of Immunoconjugates of Doxorubicin. *Bioconjug. Chem.* **1993**, *4* (6), 521–527.
3. 3. Latorre, A.; Couleaud, P.; Aires, A.; Cortajarena, A. L.; Somoza, Á. Multifunctionalization of Magnetic Nanoparticles for Controlled Drug Release: A General Approach. *Eur. J. Med. Chem.* **2014**, *82*, 355–362.
4. 4. Vitamin receptor binding drug delivery conjugates Vlahov, Iontcho Radoslavov et al From PCT Int. Appl., 2004069159, 19 Aug 2004 (https://patents.google.com/patent/WO2004069159).

© 2019 by the authors. Licensee MDPI, Basel, Switzerland. This article is an open access article distributed under the terms and conditions of the Creative Commons Attribution (CC BY) license (http://creativecommons.org/licenses/by/4.0/).
